# Supplementary material for: Frog eat frog: exploring variables influencing anurophagy
Source: PeerJ. 2015 Aug 25;3:e1204. doi: 10.7717/peerj.1204 (PMC4556157; doi:10.7717/peerj.1204)
Supplement: Supplemental Information 1 — Literature references for data used in the study. Variables extracted are: coordinates of study site; Country of study; Superfamily; family; genus and species name; whether or not the species was invasive at the study site; habitat; snout-vent length (SVL); whether there was evidence of cannibalism; the richness of amphibians at the study locality; number of individual frogs examined; proportion of anurans in diet. [file peerj-03-1204-s001.pdf]

Author	Year	Title	Journal	Volume	Issue	Pages	Country	Latitude (DD) N or -S	Longitude (DD) E or -W	Invasive Yes/No	Superfamily	Family	Genus	Species	habitat	Number in study	SVL (mean)	Cannibalism (yes/no/both)	Species richness at centroid	Proportion of prey that is amphibian
Inger, R. F., & Greenberg	1966	Ecological and compet	Ecology	47	5	746-759	Malaysia	1.633328	113.583323	no	Ranoidea	Dicroglossidae	Limnonectes	macrodon	forest	106	112	yes	48	0.105263
Cajade, R., E. F. Schaefer	2010	Trophic and microhabitat niche overlap in two		24	2	81-92	Costa Rica	10.424255	-84.022488	no	Hyloidea	Dendrobatidae	Dendrobates	auratus	forest	40	32	no	51	0
Hugo Zaracho, V., J. Luis	2012	Diet and parasitism of	Revista Mexicana De Bic	83	4	1180-1186	Argentina.	-27.316667	-58.766667	no	Hyloidea	Leptodactylidae	Adenomera	diptyx	forest	22	19.65	no	42	0
Toft, C. A.	1980	Feeding ecology of thi	Oecologia (Berl.)	45	1	131-141.	Peru	-9.583212	-74.800097	no	Hyloidea	Dendrobatidae	Allobates	femoralis	forest	24	33	no	38	0
Men,ndez-Guerrero, P	2001	Ecolog¡a tr¢fica de la c	Mongraphie, Pontificia Universidad Catolica del I				Ecuador	-1.099699	-75.80815	no	Hyloidea	Dendrobatidae	Ameerega	bilinguis	forest	22	21.22	no	104	0
Forti, L. R., A. S. O. Tissia	2011	Diet of Ameerega brac	Brazilian Journal of Biolc	71	1	189-196	Brazil	-15.4	-55.833333	no	Hyloidea	Dendrobatidae	Ameerega	braccata	forest	44	23	no	45	0
Biavati, G. M., H. C. Wiec	2004	Diet of Epipedobates f	Journal of Herpetology	38	4	510-518	Brazil	-14.209722	-47.685	no	Hyloidea	Dendrobatidae	Ameerega	flavopicta	forest	612	25	no	51	0
Lima, N. G., & P. C. Eterc	2013	Natural History of Ameerega flavopicta (Dendr		47	3	480-488	Brazil	-18.5486111	-45.3063888	no	Hyloidea	Dendrobatidae	Ameerega	flavopicta	forest	32	24	no	46	0
Toft, C. A.	1980	1980. Feeding ecology	Oecologia (Berl.)	45	1	131-141.	Peru	-9.61667	-74.93333	no	Hyloidea	Dendrobatidae	Ameerega	petersi	forest	26	32	no	32	0
Toft, C. A.	1980	Feeding ecology of thi	Oecologia (Berl.)	45	1	131-141.	Peru	-9.583212	-74.800097	no	Hyloidea	Dendrobatidae	Ameerega	trivittatus	forest	35	45	no	38	0
Gómez-Hoyos, D. A., Lóp	2014	Geographic variation i	Herpetology Notes	7		559-564.	Colombia	4.7	-75.633333	no	Hyloidea	Dendrobatidae	Andinobates	bombetes	forest	69	19	no	13	0
Toft, C. A.	1981	Ecology of Panamania	Journal of Herpetology	15	2	139-144	Panama	9.12857	-79.71588	no	Hyloidea	Dendrobatidae	Andinobates	minutus	forest	26	18	no	54	0
Valderrana-Vernaza, M.,	2009	Diet of the Andean Frc	Journal of Herpetology	43	1	114-123	Colombia	6.1	-73.216667	no	Hyloidea	Dendrobatidae	Andinobates	virolinensis	forest	166	17	no	8	0
Albertina P. Lima and Gl	1993	Effects of Prey Size an	Oecologia	95	1	93-102	Brazil	-2.887161	-59.724782	no	Hyloidea	Aromobatidae	Anomaloglossus	stepheni	forest	443	14	no	84	0
Acuña Juncá, F., P., Cabr	2007	Feeding ecology of tw	Journal of Herpetology	41	2	301-308	Brazil	-2.5	-60	no	Hyloidea	Aromobatidae	Anomaloglossus	stepheni	forest	72	16.8	no	85	0
Blackburn, D. C. and C. S	2006	Ontogenetic diet chan	Journal of Herpetology	40	3	388-394	Malawi	-15.956212	35.655283	no	Ranoidea	Arthroleptidae	Arthroleptis	xenodactyloides	forest	26	20	no	57	0
Toft, C. A.	1981	Ecology of Panamania	Journal of Herpetology	15	2	139-144	Panama	9.335996	-78.916431	no	Hyloidea	Bufonidae	Atelopus	varius	forest	23	46	no	54	0
Fontoura, P. L., L. F. Ribe	2011	Diet of Brachycephalu	Zoologia	28	5	687-689	Brazil	-25.420649	-49.228306	no	Hyloidea	Brachycephalidae	Brachycephalus	brunneus	forest	20	13	no	63	0
Almeida-Santos, M., Siqu	2011	Ecology of the Brazilian flea frog Brachycephalu		45	2	251-255	Brazil	-22.416666	-42.5833333	no	Hyloidea	Brachycephalidae	Brachycephalus	didactylus	forest	39	8.6	no	108	0
Dorigo, T. A., C. C., Sique	2012	Ecological aspects of t	Journal of Natural Histor	46		39-40	Brazil	-22.3594444	-42.567777	no	Hyloidea	Brachycephalidae	Brachycephalus	garbeanus	forest	80	15.3	no	110	0
Oliveira, E. G. D.	2013	História natural de Bra	Thesis - Instituto de Biociências do Campus de Ri				Brazil	-23.011338	-44.680286	no	Hyloidea	Brachycephalidae	Brachycephalus	pitanga	forest	30	11.75	no	97	0
Duellman, W. E. andÿM.	1994	Biology of a sit-and-wa	Herpetologica	50	1	51-64	Peru'	-12.583333	-69.08333	no	Hyloidea	Ceratophryidae	Ceratophrysÿ	cornuta	forest	72	63.47	no	82	0.015779
Van Sluys, M., G. Schittir	2006	Body size, diet and en	Brazilian Journal of Biolc	66	1A	167-173	Brazil	-17.891944	-39.371944	no	Ranoidea	Microhylidae	Chiasmocleis	capixaba	forest	119	15.7	no	62	0
Toft, C. A.	1980	Feeding ecology of thi	Oecologia (Berl.)	45	1	131-141.	Peru	-9.583212	-74.800097	no	Hyloidea	Dendrobatidae	Colostethus	marchesianus	forest	32	14.7	no	38	0
Lieberman, S. S.	1986	Ecology of the leaf litt	Acta Zool Mexicana.(Nu	15		Jan-74	Costa Rica	10.422228	-84.01931	no	Hyloidea	Craugastoridae	Craugastor	bransfordii	forest	52	17.8	no	53	0
Mart?Nez-Coronel, M. a	2011	Composici¢n de la diet	Acta zool¢gica mexicana	27	2	215-230	Mexico	17.1667	-93.15	no	Hyloidea	Craugastoridae	Craugastor	lineatus	forest	76	25.9	no	12	0
Toft, C. A.	1981	Ecology of Panamania	Journal of Herpetology	15	2	139-144	Panama	9.12857	-79.71588	no	Hyloidea	Leptodactylidae	Craugastor	talamancae	forest	27	47	no	54	0
Lieberman, S. S.	1986	Ecology of the leaf litt	Acta Zool Mexicana.(Nu	15		Jan-75	Costa Rica	10.422228	-84.01931	no	Hyloidea	Craugastoridae	Craugastor	talamancae	forest	23	28.4	no	53	0
Wachlevski, M., P. H. De	2008	Microhabitat use and	Journal of Natural Histor	42	19-2	1421-1434	Brazil	-20.09361111	-43.49972222	no	Hyloidea	Hylodidae	Crossodactylus	bokermanni	forest	48	22.7	no	77	0
Caldart V. M., S. Iop, T. R	2012	Feeding ecology of Ch	Zoological Studies	51	4	484-493	Brazil	-27.2428	-53.95381667	no	Hyloidea	Hylodidae	Crossodactylus	schmidti	forest	126	26.88	no	35	0
Men,ndez-Guerrero, P	2001	Ecolog¡a tr¢fica de la c	Mongraphie, Pontificia Universidad Catolica del I				Ecuador	-1.099699	-75.80815	no	Hyloidea	Hylidae	Dendropsophus	bifurcus	forest	25	25.37	no	104	0
Javier Méndez-Narváez1	2014	Diet and trophic ecolo	Herpetology Notes	7		299-305	Colombia	2.998352	-76.409631	no	Hyloidea	Hylidae	Dendropsophus	columbianus	forest	39	28	no	4	0
Jiménez, R., & F. Bolaños	2012	Use of food and spatia	Phyllomedusa: Journal c	11	1	51-62	Costa Rica	10.420374	-84.024437	no	Hyloidea	Hylidae	Dendropsophus	ebraccatus	forest	60	25	no	51	0
Men,ndez-Guerrero, P	2001	Ecolog¡a tr¢fica de la c	Mongraphie, Pontificia Universidad Catolica del I				Ecuador	-1.099699	-75.80815	no	Hyloidea	Hylidae	Dendropsophus	marmoratus	forest	24	38.29	no	104	0
Mayer,T. B.	2014	Caracterização do pro	Universidade Federal dc	Thesis			Brazil	-25.2858333	-49.0013888	no	Hyloidea	Hylidae	Dendropsophus	minutus	forest	107	21	no	67	0
Jiménez, R., & F. Bolaños	2012	Use of food and spatia	Phyllomedusa: Journal c	11	1	51-63	Costa Rica	10.420374	-84.024437	no	Hyloidea	Hylidae	Dendropsophus	phlebodes	forest	135	22.2	no	51	0
Men,ndez-Guerrero, P	2001	Ecolog¡a tr¢fica de la c	Mongraphie, Pontificia Universidad Catolica del I				Ecuador	-1.099699	-75.80815	no	Hyloidea	Hylidae	Dendropsophus	sarayacuensis	forest	23	26.87	no	104	0
Toft, C. A.	1981	Ecology of Panamania	Journal of Herpetology	15	2	139-144	Panama	9.335996	-78.916431	no	Hyloidea	Leptodactylidae	Diasporus	vocator	forest	35	18	no	54	0
Salvidio, S., R. Sindaco&	1999	Feeding habits of sym	Herpetological Journal	9	4	163-168	Corsica	41.680507	9.211553	no	Discoglossoidea	Alytidae	Discoglossus	montalentii	forest	22	44.4	no	5	0
Luría Manzano, R.	2012	Ecología trófica del en	Tesis - Universidad Autonoma Del Estado De Hid				Mexico	18.4	-96.845	no	Hyloidea	Hylidae	Ecnomiohyla	miotympanum	forest	74	28.9	no	10	0
Ovaska, K.	1991	Dietÿofÿtheÿfrog Eleut	Journal of Herpetology	25	4	486-488	Barbados, '	-59.631672	13.179626	no	Hyloidea	Leptodactylidae	Eleutherodactylus	johnstonei	forest	140	25	no	2	0
Men,ndez-Guerrero, P	2001	Ecolog¡a tr¢fica de la c	Mongraphie, Pontificia Universidad Catolica del I				Ecuador	-1.099699	-75.80815	no	Hyloidea	Leptodactylidae	Engystomops	petersi	forest	37	26.17	no	104	0
Reboucas, R., I. M. Castr	2013	Diet of Haddadus bino	North-Western Journal (	9	2	293-299	Brazil	-13.820917	-39.197806	no	Hyloidea	Craugastoridae	Haddadus	binotatus	forest	133	29.05	no	58	0
Coco, L., V. N. T. Borges .	2014	Feeding habits of the l	Anais da Academia Brasi	86	1	239-249	Brazil	-21	-41.233	no	Hyloidea	Craugastoridae	Haddadus	binotatus	forest	28	31.88	no	87	0
Coco, L., V. N. T. Borges .	2014	ÿFeeding habits of the	Anais da Academia Brasi	86	1	239-249	Brazil	-22.48333	-42.19667	no	Hyloidea	Craugastoridae	Haddadus	binotatus	forest	51	32.42	no	102	0
Luría Manzano, R.	2012	Ecología trófica del en	Tesis - Universidad Autonoma Del Estado De Hid				Mexico	18.4	-96.845	no	Hyloidea	Centrolenidae	Hyalinobatrachium	fleischmanni	forest	59	23.1	no	10	0
Almeida-Gomes, M., F. H	2007	Diet and microhabitat	Iheringia Serie Zoologia	97	1	27-30	Brazil	-23.183333	-44.2	no	Hyloidea	Cycloramphidae	Hylodes	phyllodes	forest	48	33.9	no	17	0
Lieberman, S. S.	1986	Ecology of the leaf litt	Acta Zool Mexicana.(Nu	15		Jan-76	Costa Rica	10.422228	-84.01931	no	Ranoidea	Microhylidae	Hypopachus	pictiventris	forest	24	27.8	no	53	0
Telles D. O. C.,ÿ S. A. F. V	2013	Reproductive biology,	Phyllomedusa	12	1	69-76	Brazil	-3.076	-59.958	no	Hyloidea	Hylidae	Hypsiboas	cinerascens	forest	23	32.45	no	78	0
Men,ndez-Guerrero, P	2001	Ecolog¡a tr¢fica de la c	Mongraphie, Pontificia Universidad Catolica del I				Ecuador	-1.099699	-75.80815	no	Hyloidea	Hylidae	Hypsiboas	lanciformis	forest	26	73.48	no	104	0
Munoz-Guerrero, J., V. H	2007	Microhabitat use, diet	Caldasia	29	2	413-425	Colombia	9	-73.966667	no	Hyloidea	Hylidae	Hypsiboas	pugnax	forest	31	53	no	22	0
L¢pez, J. A., Scarabotti, F	2009	Is the red spotted gree	Revista de biolog¡a trop	57	3	847-857	Argentina	-60.666667	-31.633333	no	Hyloidea	Hylidae	Hypsiboas	punctatus	forest	47	26.07446809	no	30	0
Smith, G. R., J. A. Lemos-	2011	Diets of three species	Western North America	71	4	563-569	Mexico	28.3057	-109.3005	no	Hyloidea	Bufonidae	Incilius	mazatlanensis	forest	21	61.8	no	9	0
Dietl, J., W. Engels and N	2009	Diet and feeding beha	Journal of Natural Histor	43	23-2	1473-1483	USA	-29.55	-50.21666	no	Hyloidea	Brachycephalidae	Ischnocnema	henselii	forest	79	35	no	41	0
Dietl, J., W. Engels and N	2009	Diet and feeding beha	Journal of Natural Histor	43	23-2	1473-1483	Brazil	-19.816667	-43.95	no	Hyloidea	Brachycephalidae	Ischnocnema	juipoca	forest	115	23	no	61	0
Martins, A. C. J., M. C. Ki	2010	Ecology of Ischnocnem	Zoologia (Curitiba)	27	2	201-208	Brazil	-22.366666	-43.797777	no	Hyloidea	Brachycephalidae	Ischnocnema	parva	forest	240	14.1	no	96	0
Brooks, GR	1982	An Analysis of Prey Co	biotropica	14	4	301-309	West Indie	15.424601	-61.377282	no	Hyloidea	Leptodactylidae	Leptodactylus	fallax	forest	371	98	no	1	0.03114

Javier Méndez-Narváez1	2014	Diet and trophic ecolo	Herpetology Notes	7		299-305	Colombia	2.998352	-76.409631	no	Hyloidea	Leptodactylidae	Leptodactylus	fragilis	forest	45	40	no	4	0
Enabulele, E. E., & M. S.	2012	Diets of Hemisus mam	Zoologist	10		48-53	Nigeria	6.111486	5.300583	no	Ranoidea	Arthroleptidae	Leptopelis	spiritusnoctis	forest	51	28.2	no	31	0
Inger, R. F., & Greenberg	1966	Ecological and compet	Ecology	47	5	746-759	Malaysia	1.633328	113.583323	no	Ranoidea	Dicroglossidae	Limnonectes	blythii	forest	445	110	no	48	0.011429
Inger, R. F.	1969	Organization of comm	The Journal of Animal Ec	38	1	, 123-148.	Malaysia	1.616811	113.583355	no	Ranoidea	Dicroglossidae	Limnonectes	blythii	forest	235	110	no	48	0.001905
Inger, R. F., & Greenberg	1966	Ecological and compet	Ecology	47	5	746-759	Malaysia	1.633328	113.583323	no	Ranoidea	Dicroglossidae	Limnonectes	ibanorum	forest	85	100	no	48	0
Inger, R. F.	1969	Organization of comm	The Journal of Animal Ec	38	1	, 123-148.	Malaysia	1.616811	113.583355	no	Ranoidea	Dicroglossidae	Limnonectes	ibanorum	forest	50	100	no	48	0
Inger, R. F.	1969	Organization of comm	The Journal of Animal Ec	38	1	, 123-148.	Malaysia	1.616811	113.583355	no	Ranoidea	Dicroglossidae	Limnonectes	macrodon	forest	50	112	no	48	0.037594
Hodgkison, S. and J. M. H	2003	Seasonal, sexual and o	Wildlife Research	30	4	345-354	Australia	-17.766667	145.583333	no	Hyloidea	Hylidae	Litoria	nannotis	forest	298	55	no	41	0
Hodgkison, S. and J. M. H	2003	Seasonal, sexual and o	Wildlife Research	30	4	345-354	Australia	-17.766667	145.583333	no	Hyloidea	Hylidae	Litoria	rheocola	forest	121	35	no	41	0
Bortolini, S. V., R. Maney	2013	Diet of Melanophrynis	Herpetological Journal	23	2	115-119	Brazil	-28.042	-51.224	no	Hyloidea	Bufonidae	Melanophryniscus	devincenzii	forest	46	24.5	no	38	0
Fernanda Quiroga, M., M	2011	Population diet variati	Amphibia-Reptilia	32	2	261-265	Argentina	-22.266667	-64.683333	no	Hyloidea	Bufonidae	Melanophryniscus	rubriventris	forest	138	37	no	12	0
Bonansea, M. I. and M. V	2007	Geographic variation c	Journal of Herpetology	41	2	231-236	Argentina	-22.2667	-64.6833	no	Hyloidea	Bufonidae	Melanophryniscus	rubriventris	forest	132	37	no	5	0
Kam, Y. C., T. C. Chen, J.	1998	Seasonal activity, repr	Journal of Herpetology	32	3	447-452	Taiwan	24.090778	121.027949	no	Ranoidea	Ranidae	Odorrana	swinhoana	forest	69	90	no	21	0
Arce Dom?nguez, F. and	2013	Dieta de Phyllobates a	Acta zool¢gica mexicana	29	2	255-268	Colombia	5.525833	-76.995	no	Hyloidea	Dendrobatidae	Oophaga	histrionica	forest	20	31.5	no	50	0
Arce Domínguez, F. & Re	2013	Dieta de Phyllobates a	Acta zoológica mexicana	29	2	255-269	Colombia	5.5258333	-76.995	no	Hyloidea	Dendrobatidae	Oophaga	histrionica	forest	20	39	no	50	0
Osorio-Domínguez, D., L	2015	Descripción de la dieta	Rev. Biodivers. Neotrop.	5	1	29-35	Colombia	3.785654	-76.719511	no	Hyloidea	Dendrobatidae	Oophaga	histrionica	forest	46	39	no	34	0
Cajade, R., E. F. Schaefer	2010	Trophic and microhabitat niche overlap in two		24	2	81-93	Costa Rica	10.424255	-84.022488	no	Hyloidea	Dendrobatidae	Oophaga	pumilio	forest	40	21	no	51	0
Lieberman, S. S.	1986	Ecology of the leaf litt	Acta Zool Mexicana.(Nu	15		Jan-72	Costa Rica	10.422228	-84.01931	no	Hyloidea	Dendrobatidae	Oophaga	pumilio	forest	26	20.1	no	53	0
Men,ndez-Guerrero, P	2001	Ecolog¡a tr¢fica de la c	Mongraphie, Pontificia Universidad Catolica del I				Ecuador	-1.099699	-75.80815	no	Hyloidea	Hylidae	Osteocephalus	planiceps	forest	33	56.91	no	104	0
Men,ndez-Guerrero, P	2001	Ecolog¡a tr¢fica de la c	Mongraphie, Pontificia Universidad Catolica del I				Ecuador	-1.099699	-75.80815	no	Hyloidea	Hylidae	Osteocephalus	yasuni	forest	28	52.34	no	104	0
Inger, R. F.	1969	Organization of comm	The Journal of Animal Ec	38	1	, 123-148.	Malaysia	1.616811	113.583355	no	Ranoidea	Ranidae	Philautus	hosii	forest	24	75	no	48	0
Inger, R. F.	1969	Organization of comm	The Journal of Animal Ec	38	1	, 123-148.	Malaysia	1.616811	113.583355	no	Hyloidea	Bufonidae	Phrynoidis	asper	forest	25	105	no	48	0
Arce Dom?nguez, F. and	2013	Dieta de Phyllobates a	Acta zool¢gica mexicana	29	2	255-268	Colombia	5.525833	-76.995	no	Hyloidea	Dendrobatidae	Phyllobates	aurotaenia	forest	20	32	no	50	0
Arce Domínguez, F. & Re	2013	Dieta de Phyllobates a	Acta zoológica mexicana	29	2	255-268	Colombia	5.5258333	-76.995	no	Hyloidea	Dendrobatidae	Phyllobates	aurotaenia	forest	20	34	no	50	0
de Paula Lima, J. E., D. Ro	2010	Diet of two sympatric	North-Western Journal o	6	1	13-24	Brazil	-14.796	-39.172	no	Hyloidea	Hylidae	Phyllomedusa	burmeisteri	forest	99	72	no	65	0
Arroyo, S. B., V. H. Serran	2008	Diet, microhabitat and	Phyllomedusa: Journal o	7	2	109-119	Columbia	7.05131	-72.95	no	Ranoidea	Strabomantidae	Pristimantis	miyatai	forest	59	20	no	9	0
R”dder, D.	2011	Diet of Physalaemus b	Russian Journal of Herpe	15	2	117-121	Bolivia	-16.642498	-65.561186	no	Hyloidea	Craugastoridae	Pristimantis	platydactylus	forest	58	26.5	no	20	0
Leonardo Boquimpani-Fr	2002	Ecology of the Horned	Leaf-Frog, Proceratophr	36	2	318-322	Brazil	-23.165407	-44.21871	no	Hyloidea	Leptodactylidae	Proceratophrys	appendiculata	forest	18	59.1375	no	17	0.055556
Klaion, T., M. Almeida-Go	2011	Diet and nematode inf	Anais Da Academia Bras	83	4	1303-1312	Brazil	-22.483	-42.917	no	Hyloidea	Cycloramphidae	Proceratophrys	boiei	forest	38	57	no	114	0
Neves, M. D. O., Morais,	2014	Sexual Dimorphism an	South American Journal	9	3	177-182.	Brazil	-6.776302	-45.540685	no	Hyloidea	Hylidae	Pseudis	tocantins	forest	184	45	no	32	0
Pengilley R. K.	1971	The food of some Aust	Journal of Zoology				Australia	-35.289	145.503	no	Hyloidea	Myobatrachidae	Pseudophryne	dendyi	forest	54	27	no	11	0
Luría Manzano, R.	2012	Ecología trófica del en	Tesis - Universidad Autonoma Del Estado De Hid				Mexico	18.4	-96.845	no	Hyloidea	Hylidae	Ptychohyla	zophodes	forest	69	33.2	no	10	0
Kam, Y. C., C. S. Wang ar	1995	REPRODUCTION AND I	Zoological Studies	34	3	193-201	Taiwan	24.999189	121.87099	no	Ranoidea	Ranidae	Rana	longicrus	forest	148	55	no	19	0
Sabagh, L. T., & A. M. Ca	2008	Feeding overlap in two	Revista Brasileira de Zoo	25	2	247-254	Brazil	-22.467936	-42.992265	no	Hyloidea	Bufonidae	Rhinella	crucifer	forest	30	69.7	no	123	0
Ferreira R. B. & R. L Teixe	2009	Feeding patterns and u	Act Herpetologica	4	2	125-134	Brazil	-20.799563	-40.655844	no	Hyloidea	Bufonidae	Rhinella	crucifer	forest	61	70.1	no	73	0
de Mattos Brito, L. B.,ÿF.	2013	Diet, activity patterns,	The Herpetological Jour	23	1	29-37	Brazil	-4.266666	-38.933333	no	Hyloidea	Bufonidae	Rhinella	hoogmoedi	forest	145	19.87	no	30	0
Men,ndez-Guerrero, P	2001	Ecolog¡a tr¢fica de la c	Mongraphie, Pontificia Universidad Catolica del I				Ecuador	-1.099699	-75.80815	no	Hyloidea	Bufonidae	Rhinella	margaritifera	forest	25	53.69	no	104	0
Maia-Carneiro T., M. C. K	2013	Feeding habits, microh	North-Western Journal o	9	1	157-165	Brazil	-22.483333	-42.9	no	Hyloidea	Bufonidae	Rhinella	ornata	forest	39	49.412	no	114	0
Maragno, F. P. and F. L. S	2011	Diet of Rhinella scitula	Revista Mexicana De Bio	82	3	879-886	Brazil	-20.680278	-56.888056	no	Hyloidea	Bufonidae	Rhinella	scitula	forest	99	40	no	43	0
Weslei Pertel, Rogério L.	2010	Comparación de la die	Caldasia	32	1	149-159	Brazil	-20.066642	-41.033355	no	Hyloidea	Hylidae	Scinax	arduous	forest	21	18	no	85	0
Toft, C. A.	1981	Ecology of Panamanian	Journal of Herpetology	15	2	139-144	Panama	9.12857	-79.71588	no	Hyloidea	Dendrobatidae	Silverstoneia	flotator	forest	27	16	no	54	0
Toft, C. A.	1981	Ecology of Panamanian	Journal of Herpetology	15	2	139-144	Panama	9.335996	-78.916431	no	Hyloidea	Dendrobatidae	Silverstoneia	nubicola	forest	30	19	no	54	0
Lieberman, S. S.	1986	Ecology of the leaf litt	Acta Zool Mexicana.(Nu	15		Jan-73	Costa Rica	10.422228	-84.01931	no	Hyloidea	Craugastoridae	Strabomantis	biporcatus	forest	21	25.4	no	53	0
Siqueira, C. C.,ÿM.ÿVan S	2006	Feeding ecology of Th	Journal of Herpetology	40	4	520-525	Brazil	-19.4	-40.53333	no	Hyloidea	Cycloramphidae	Thoropa	miliaris	forest	22	42.6	no	73	0
Siqueira, C. C.,ÿM.ÿVan S	2006	Feeding ecology of Th	Journal of Herpetology	40	4	520-525	Brazil	-23.1833333	-44.2	no	Hyloidea	Cycloramphidae	Thoropa	miliaris	forest	33	44.5	no	17	0
Siqueira, C. C.,ÿM.ÿVan S	2006	Feeding ecology of Th	Journal of Herpetology	40	4	520-525	Brazil	-22.35	-43.783333	no	Hyloidea	Cycloramphidae	Thoropa	miliaris	forest	27	47.7	no	92	0
Weslei Pertel, Rogério L.	2010	Comparación de la die	Caldasia	32	1	149-159	Brazil	-20.066642	-41.033355	no	Hyloidea	Cycloramphidae	Thoropa	miliaris	forest	28	29	no	85	0
Toft, C. A.	1981	Ecology of Panamanian	Journal of Herpetology	15	2	139-144	Panama	9.335996	-78.916431	no	Hyloidea	Bufonidae	Trachycephalus	typhonius	forest	48	103	no	54	0
Van Sluys, M., C. F. D. Ro	2001	Diet, reproduction, an	Journal of Herpetology	35	2	322-325	Brazil	-23.18216667	-44.21386111	no	Hyloidea	Leptodactylidae	Zachaenus	parvulus	forest	70	20.3	no	17	0
Vrcibradic, D., R. L. Teixe	2009	Sexual dimorphism, re	Journal of Natural Histor	43	35-3	2245-2256	Brazil	-19.966667	-40.533333	no	Hyloidea	Hylidae	Itapotihyla	langsdorffii	forest	37	88.79	possible	85	0.12
Judith Piñero Bonilla1 y I	1996	Hábitos ??limentarios	REv Biol Trop	44	2	827-833	Venezuela	8.616943	-71.150032	no	Hyloidea	Dendrobatidae	Nephelobates	alboguttatus	forest	149	25	possible	8	0.00239
Fran‡a, L., K.ÿFacure,ÿan	2004	Trophic and spatial nic	Studies on Neotropical F	39	3	243-248	Brazil	-18.916666	-48.2833333	no	Hyloidea	Leptodactylidae	Leptodactylus	labyrinthicus	forest	57	165	yes	50	0.027027
Van Ngo, B. and C. D.ÿNg	2014	Variation in dietary co	The Herpetological Jour	24	4	245-253	Vietnam	16.166666	107.5	no	Ranoidea	Dicroglossidae	Quasipaa	verrucospinosa	forest	539	91.14	yes	29	0.00189
Brasileiro, C. A., M. Mart	2010	Feeding ecology of Th	South American Journal	5	3	181-188	Brazil	-23.495	-45.147777	no	Hyloidea	Cycloramphidae	Thoropa	taophora	forest	356	44.8	yes	73	0
Beard, K. H.	2007	Diet of the invasive fro	Copeia		2	281-291	Hawaii	19.854983	-155.15315	yes	Hyloidea	Leptodactylidae	Eleutherodactylus	coqui	forest	696	45	yes	3	0.000719
Olson, C. A. and K. H. Bea	2012	Diet of the Introduced	Copeia		1	121-129	Hawaii (US	19.576	-155.1408	yes	Hyloidea	Leptodactylidae	Eleutherodactylus	planirostris	forest	427	20	yes	3	0.000267
Bogdan, H. V., S. D. Cova	2013	How do we share food	Animal Biodiversity and	36		86-100	Romania	45.45729	22.445	no	Ranoidea	Ranidae	Pelophylax	ridibundus	generalist	110	80.1	possible	6	0
Bruce K. Johnson and Jar	1976	ReptilesThe Food and	Journal of Herpetology	10	2	63-74	USA	41.311816	-94.003389	no	Hyloidea	Hylidae	Acris	blanchardi	generalist	218	22	no	8	0
Labanick GM	1976	Prey availability, consu	J. Herpetol	10		293-298	USA	39.464622	-87.465026	no	Hyloidea	Hylidae	Acris	crepitans	generalist	279	24	no	14	0
Smith, C. C., & A. N. Brag	1949	Observations on the e	Ecology	30	3	333-349	USA	35.164006	-97.207129	no	Hyloidea	Bufonidae	Anaxyrus	americanus	generalist	165	73	no	15	0
Campbell, J. B.	1970	Food habits of the bor	Journal of Herpetology	4	###	83-85	USA	40.063888	-105.5666	no	Hyloidea	Bufonidae	Anaxyrus	boreas	generalist	33	85	no	7	0
Smith, C. C., & A. N. Brag	1949	Observations on the e	Ecology	30	3	333-351	USA	35.164006	-97.207129	no	Hyloidea	Bufonidae	Anaxyrus	cognatus	generalist	435	100	no	15	0

Smith, G. R., J. A. Lemos-	2011	Dietsÿofÿthreeÿspecie	Western North America	71	4	563-569	Mexico	27.0291	-103.10667	no	Hyloidea	Bufonidae	Anaxyrus	debilis	generalist	32	46	no	7	0
Clarke, R. D.	1974	Food habits of toads, genus Bufo (Amphibia: Bu		91	1	140-147	USA	41.375253	-72.988937	no	Hyloidea	Bufonidae	Anaxyrus	fowleri	generalist	108	60	no	11	0
Bush F. M. and E. F. Men	1962	The Food of Bufo woo	Herpetologica	18	2	110-114	USA	34.01509	-83.378799	no	Hyloidea	Bufonidae	Anaxyrus	fowleri	generalist	111	60	no	15	0
Smith, G. R., J. A. Lemos-	2011	Diets of three species	Western North America	71	4	563-569	Mexico	28.36778	-108.9257	no	Hyloidea	Bufonidae	Anaxyrus	punctatus	generalist	23	60	no	7	0
Smith, C. C., & A. N. Brag	1949	Observations on the e	Ecology	30	3	333-350	USA	35.164006	-97.207129	no	Hyloidea	Bufonidae	Anaxyrus	woodhousii	generalist	517	90	no	15	0
Dan Coga' lniceanu1, Mi	2000	Feeding in anuran com	Amphibia-Reptilia	22			Romania	45.166667	27.933333	no	Discoglossoidea	Bombinatoridae	Bombina	bombina	generalist	142	41	no	9	0
Mollov, I. A. and A. M. St	2010	Dietÿÿandÿtrophicÿnic	Biotechnology & Biotech	24	2	263-269	Poland	54.456528	18.208961	no	Hyloidea	Bufonidae	Bombina	bombina	generalist	37	41	no	11	0
Széplaki, E., I. aszalós, N.	2006	Feeding niche characte	Analele Universit??ii din	13		14-17	Romania	47.886985	23.14009	no	Discoglossoidea	Bombinatoridae	Bombina	bombina	generalist	87	41	no	8	0
Feren?i, S., L., Citrea, H.	2007	Analiza comparativ? a	Biharean Biologist	1		15-22	Romania	47.942113	23.211312	no	Discoglossoidea	Bombinatoridae	Bombina	bombina	generalist	102	41	no	8	0
Groza M. I.,ÿI. C. Suciu, M	2008	Comparison between	Biharean Biologist	2		50-57	Romania	46.905018	22.490832	no	Discoglossoidea	Bombinatoridae	Bombina	variegata	generalist	178	42	no	8	0
Cicort-Lucaciu, A. S., D. C	2011	Feeding of two amphil	North-Western Journal o	7	2	297-303	Romania	46.939333	22.522273	no	Discoglossoidea	Bombinatoridae	Bombina	variegata	generalist	85	44.71	no	8	0
Bogdan, H. V., S. D. Cova	2013	How do we share food	Animal Biodiversity and	36		86-99	Romania	45.45729	22.445	no	Discoglossoidea	Bombinatoridae	Bombina	variegata	generalist	82	44.71	no	6	0
Ghiurc?, D., & L., Zaharia	2005	Data regarding the tro	North Western Journal o	1		15-24	Romania	46.587274	26.75036	no	Hyloidea	Bombinatoridae	Bombina	variegata	generalist	57	44.7	no	9	0
Ghiurc?, D., L., Zaharia, L	2005	Data regarding the tro	Insecta–total	113	90	4	Romania	46.956567	26.333023	no	Discoglossoidea	Bombinatoridae	Bombina	variegata	generalist	27	44.7	no	9	0
Ghiurc?, D. & I. Zaharia	2006	Data regarding the tro	Complexul Muzeal de ?t	21	4	31-36	Romania	46.149428	26.447416	no	Discoglossoidea	Bombinatoridae	Bombina	variegata	generalist	70	44.7	no	5	0
Bisa, R., S., Sfenthourakis	2007	Population density and	Journal of Biological Res	8		129-137	Greece	39.919505	21.260086	no	Discoglossoidea	Bombinatoridae	Bombina	variegata	generalist	37	44.7	no	8	0
Bisa, R., S., Sfenthourakis	2007	Population density and	Journal of Biological Res	8		129-137	Greece	39.941188	21.042795	no	Discoglossoidea	Bombinatoridae	Bombina	variegata	generalist	29	41.7	no	8	0
Hodi?an, O., D. Cup?a, N	2002	The trophic spectrum	Herpetologica Romanica	3		57-61	Romania	44.984422	26.430505	no	Discoglossoidea	Bombinatoridae	Bombina	variegata	generalist	39	44.7	no	8	0
Crnobrnja-Isailovic, J., S.	2012	Diet Composition and	Journal of Herpetology	46	4	562-567	Serbia	44.689444	20.516777	no	Hyloidea	Bufonidae	Bufo	Bufo	generalist	142	77.17	no	8	0
Crnobrnja-Isailovic, J., S.	2012	Diet Composition and	Journal of Herpetology	46	4	562-567	Serbia	44.69031	20.51727	no	Hyloidea	Bufonidae	Bufo	Bufo	generalist	142	110	no	19	0
Mollov, I. A. and A. M. St	2010	Diet and trophic niche	Biotechnology & Biotech	24	2	263-269	Poland	54.456528	18.208961	no	Hyloidea	Bufonidae	Bufo	bufo	generalist	20	107	no	11	0
Gittins, S. P. (1987).	1987	The diet of the commo	Amphibia-Reptilia	8		13-17	UK	52.179552	-3.5482	no	Hyloidea	Bufonidae	Bufo	bufo	generalist	161	107	no	2	0
Wu, Z., Y. Li and Y. Wang	2007	A comparison of stoma	Acta Zoologica Sinica	53	2	364-372	China	29.843852	122.032164	no	Hyloidea	Bufonidae	Bufo	gargarizans	generalist	43	78.26	no	13	0
Yu, T., & Guo, Y.	2012	Trophic ecology and m	Zoologia (Curitiba)	29	1	54-58	China	30.583333	105.75	no	Hyloidea	Bufonidae	Bufo	gargarizans	generalist	49	97.3	no	10	0
Yu, T. L., Y. S. Gu, J. Du &	2009	Seasonal variation and	Biharean Biologist	3	2	99-104	China	30.58333	105.75	no	Hyloidea	Bufonidae	Bufo	gargarizans	generalist	109	80	no	10	0
Hirai, T., & M. Matsui	2002	Feeding ecology of Bufo japonicus formosus fro		36	4	719-723	Japan	35.117028	135.758472	no	Hyloidea	Bufonidae	Bufo	japonicus	generalist	55	148.5	no	13	0
Covaciu-Marcov S.D., S.	2012	Terrestrial isopods in the diet of two amphibian		17		11-May	Romania	44.759735	28.724258	no	Hyloidea	Bufonidae	Bufotes	viridis	generalist	21	85	no	9	0
Covaciu-Marcov, S. D., D	2010	Human influence or na	Acta Zoologica Bulgarica	62	3	307-316	Romania	44.775114	28.890118	no	Hyloidea	Bufonidae	Bufotes	viridis	generalist	34	74.3	no	9	0
Araujo, M. S., D. I. Bolnic	2009	Individual-level diet va	Journal of Animal Ecolog	78	4	848-856	Brazil	-18.92	-48.285	no	Ranoidea	Microhylidae	Chiasmocleis	albopunctata	generalist	51	27.7	no	50	0
Mac Nally, R. C.	1983	Trophic relationships o	Herpetologica	39	2	130-140	Australia	-37.28277	144.966431	no	Hyloidea	Myobatrachidae	Crinia	parinsignifera	generalist	77	22	no	13	0
Pengilley R. K.	1971	The food of some Aust	Journal of Zoology				Australia	-35.289	145.503	no	Hyloidea	Myobatrachidae	Crinia	signifera	generalist	46	23	no	11	0
Mac Nally, R. C.	1983	Trophic relationships o	Herpetologica	39	2	130-140	Australia	-37.28277	144.966431	no	Hyloidea	Myobatrachidae	Crinia	signifera	generalist	148	30	no	13	0
Munoz-Guerrero, J., V. H	2007	Microhabitat use, diet	Caldasia	29	2	413-425	Colombia	9	-73.966667	no	Hyloidea	Hylidae	Dendropsophus	microcephalus	generalist	54	26	no	22	0
Salvidio, S., R. Sindaco&	1999	Feeding habits of symp	Herpetological Journal	9	4	163-167	Corsica	41.680507	9.211553	no	Discoglossoidea	Alytidae	Discoglossus	sardus	generalist	49	48.4	no	5	0
Berry P. Y and J. A. Bullo	1962	The food of the comm	Copeia	###	4	736-741	Singapore	1.317649	103.816168	no	Hyloidea	Bufonidae	Duttaphrynus	melanostictus	generalist	109	79.1	no	25	0
Berry P. Y and J. A. Bullo	1962	The food of the comm	Copeia	###	4	736-742	Malaysia	3.13875	101.791203	no	Hyloidea	Bufonidae	Duttaphrynus	melanostictus	generalist	30	79.1	no	41	0
Norval, G., S. C. Huang, J	2014	Notes on the diets of five amphibian species fro		30		69-77	Taiwan	23.43283333	120.485	no	Hyloidea	Bufonidae	Duttaphrynus	melanostictus	generalist	77	26.4	no	15	0
Pinto, P. M.	2011	Ecologia alimentar de	Thesis - Universidade de Brasilia				Brazil	-15.571951	-47.52666	no	Ranoidea	Microhylidae	Elachistocleis	cesarii	generalist	171	28.6	no	54	0
Dean B. Premo and Anita	1987	Dietary Patterns of the	Herpetologica	43	1	1 to 6	Indonesia	-6.608324	106.790519	no	Ranoidea	Dicroglossidae	Fejervarya	cancrivora	generalist	326	79	no	19	0.035639
Wu, Z., Y. Li and Y. Wang	2007	A comparison of stoma	Acta Zoologica Sinica	53	2	364-372	China	29.843852	122.032164	no	Ranoidea	Dicroglossidae	Fejervarya	limnocharis	generalist	174	41.04	no	13	0
Yu, T., & Guo, Y.	2012	Trophic ecology and m	Zoologia (Curitiba)	29	1	54-60	China	30.583333	105.75	no	Ranoidea	Dicroglossidae	Fejervarya	limnocharis	generalist	35	36.2	no	10	0
Deyrup, M., L. Deyrup &	2013	Ant species in the diet	Southeastern Naturalist	12	2	367-378	USA	27.186744	-81.34741	no	Ranoidea	Microhylidae	Gastrophryne	carolinensis	generalist	146	25	no	16	0
Hirai, Toshiaki; Matsui, M	2000	Myrmecophagy in a ra	Zoological Science	17	4	459-466	Japan	35.10054	135.86627	no	Ranoidea	Ranidae	Glandirana	rugosa	generalist	72	38	no	12	0
Enabulele, E. E., & M. S.	2012	Diets of Hemisus mam	Zoologist	10		48-52	Nigeria	6.111486	5.300583	no	Ranoidea	Hemisotidae	Hemisus	marmoratus	generalist	22	29.9	no	31	0
Sabagh, L. T., V. L. Ferrei	2010	Living together, somet	Brazilian Journal of Biolo	70	4	955-960	Brazil	-19.56666	-57	no	Hyloidea	Hylidae	Hipsoboas	raniceps	generalist	37	44.8	no	43	0
Dan Coga' lniceanu1, Mi	2000	Feeding in anuran com	Amphibia-Reptilia	22			Romania	45.166667	27.933333	no	Hyloidea	Hylidae	Hyla	arborea	generalist	34	45	no	9	0
Lur¡a-Manzano, R. and G	2014	Reproduction and diet	Vertebrate Zoology	64	2	207-213	Mexico	18.283333	-97.066667	no	Hyloidea	Hylidae	Hyla	euphorbiaceae	generalist	36	36.05	no	9	0
Hirai, T.	2007	Diet composition of Hy	Japanese Journal of App	51	2	103-106	Japan	38.569643	140.972329	no	Hyloidea	Hylidae	Hyla	japonica	generalist	101	33	no	10	0
Mahan, R. D. and J. R. Jo	2007	Diet of the Gray Treefr	Journal of Herpetology	41	1	16-23	Missouri, U	38.771909	-92.197695	no	Hyloidea	Hylidae	Hyla	versicolor	generalist	107	41.5	no	16	0
Yu, T., & Guo, Y.	2012	Trophic ecology and m	Zoologia (Curitiba)	29	1	54-59	China	30.583333	105.75	no	Ranoidea	Ranidae	Hylarana	guentheri	generalist	25	56.3	no	10	0
Men,ndez-Guerrero, P	2001	Ecolog¡a tr¢fica de la c	Mongraphie, Pontificia Universidad Catolica del I				Ecuador	-1.099699	-75.80815	no	Hyloidea	Hylidae	Hypsiboas	geographicus	generalist	27	52.21	no	104	0
Da Rosa, I. , A. Canavero	2011	Trophic niche variation	South American Journal	6	2	98-106	Uruguay	-34.783333	-55.36666	no	Hyloidea	Hylidae	Hypsiboas	pulchellus	generalist	169	105	no	19	0
Maneyro, R. and I. da Ro	2004	Temporal and spatial c	Phyllomedusa: Journal c	3	2	101-103	Uruguay	-34.783333	-55.366667	no	Hyloidea	Hylidae	Hypsiboas	pulchellus	generalist	105	50	no	19	0
Arau´jo MS, Dos Reis SF,	2007	Intrapopulation diet va	Copeia	###		855-865	Brazil	-18.916763	-48.283816	no	Hyloidea	Leptodactylidae	Leptodactylus	cf. andreae	generalist	100	21	no	50	0
Massao Moreira Sugai, J	2012	Diet of Leptodactylus f	Biota Neotropica	12	1	99-104	Brazil	-19.576944	-57.011667	no	Hyloidea	Leptodactylidae	Leptodactylus	fuscus	generalist	33	41.48	no	43	0
Sugai, J. L. M. M., J. d. S.	2012	Diet of Leptodactylus f	Biota Neotropica	12	1	99-104	Brazil	-19.576944	-57.011667	no	Hyloidea	Leptodactylidae	Leptodactylus	fuscus	generalist	44	41.48	no	43	0
Arau´jo MS, Dos Reis SF,	2007	Intrapopulation diet va	Copeia	###		855-865	Brazil	-18.916763	-48.283816	no	Hyloidea	Leptodactylidae	Leptodactylus	fuscus	generalist	83	35	no	50	0.002252
Sanabria, E. A., L. B. Quir	2005	Dieta de Leptodactylus	Revista Peruana de Biolo	12	3	473-477	Argentina	-31.1966666	-69.1666666	no	Hyloidea	Leptodactylidae	Leptodactylus	latrans	generalist	35	43.69	no	1	0.051282
Strussmann, C., M. B. R.	1984	Diet and foraging mod	Journal of Herpetology	18	2	138-146	Brazil	-2.476180556	-54.95842222	no	Hyloidea	Leptodactylidae	Leptodactylus	latrans	generalist	21	70	no	79	0
Teixeira, R. L. and D. Vrci	2003	Diet of Leptodactylus c	Cuadernos de Herpetolo	17	1	111-118	Brazil	-20.766667	-40.583333	no	Hyloidea	Leptodactylidae	Leptodactylus	latrans	generalist	57	70	no	74	0.027778
Pinto, P. M.	2011	Ecologia alimentar de	Thesis - Universidade de Brasilia				Brazil	-15.571951	-47.52666	no	Hyloidea	Leptodactylidae	Leptodactylus	latrans	generalist	21	110	no	54	0
Lajmanovic R. C.	1996	Dinamica trophica de j	Cuadernos de Herpetolo	10			Argentina	-31.68333	-60.7	no	Hyloidea	Leptodactylidae	Leptodactylus	macrosternum	generalist	31	80	no	30	0

Ferreira, R. B., R. B. Dant	2007	Reproduction and ont	Boletim do Museu de Bi	22		45-55	Brazil	-20.756459	-40.574289	no	Hyloidea	Leptodactylidae	Leptodactylus	natalensis	generalist	103	39	no	74	0.026144
Rodrigues, D. d. J., M. Ue	2004	Seasonal and ontogen	Rev. Esp. Herp	18		19-28	Brazil	-19.56666667	-57	no	Hyloidea	Leptodactylidae	Leptodactylus	podicipinus	generalist	189	29.63	no	43	0
Parker, M. L. and M. I. G	2004	Diet of the Rio Grande	Journal of Herpetology	38	1	127-130	Texas, USA	26.755575	-98.243519	no	Ranoidea	Ranidae	Lithobates	berlandieri	generalist	85	18	no	19	0
Ramirez-Bautista, A. and	2004	Diets of two syntopic	Southwestern Naturalist	49	3	316-320	Mexico	18.7	-95.47	no	Ranoidea	Ranidae	Lithobates	brownorum	generalist	22	50.8	no	19	0
Aurelio Ramírez-Bautista	2004	Diets of Two Syntopic	The Southwestern Natu	49	3	316-320.	Mexico	18.599725	-95.066926	no	Ranoidea	Ranidae	Lithobates	brownorum	generalist	22	55	no	20	0
Lamb, T.	1984	THE INFLUENCE OF SE	American Midland Natu	111	2	311-318	USA	31.452421	-84.364498	no	Ranoidea	Ranidae	Lithobates	grylio	generalist	122	110	no	24	0.00565
Hedeen SE	1972	Food and feeding beh	Am Midl Nat	88		291-300	USA	47.214097	-95.22927	no	Ranoidea	Ranidae	Lithobates	pipiens	generalist	23	62	no	9	0
Moore, J. E., & Stricklanc	1954	Notes on the food of t	American Midland Natu	52	1	221-224	Canada	51.378093	-109.996743	no	Ranoidea	Ranidae	Lithobates	pipiens	generalist	20	60	no	4	0
Hedeen SE	1972	Food and feeding beh	Am Midl Nat	88		291-300	USA	47.214097	-95.22927	no	Ranoidea	Ranidae	Lithobates	septentrionalis	generalist	60	56	no	9	0
Crawford, J. A., D. B. She	2009	Diet Composition and	Copeia		4	642-646	USA	35.75	-95.15	no	Ranoidea	Ranidae	Lithobates	sphenocephalus	generalist	62	31.16	no	16	0
Ramirez-Bautista, A. and	2004	Diets of two syntopic	Southwestern Naturalist	49	3	316-320	Mexico	18.7	-95.47	no	Ranoidea	Ranidae	Lithobates	vaillanti	generalist	26	74.7	no	19	0
Aurelio Ramírez-Bautista	2004	Diets of Two Syntopic	The Southwestern Natu	49	3	316-320.	Mexico	18.599725	-95.066926	no	Ranoidea	Ranidae	Lithobates	vaillanti	generalist	26	75	no	20	0
Pengilley R. K.	1971	The food of some Aust	Journal of Zoology				Australia	-35.289	145.503	no	Hyloidea	Hylidae	Litoria	verreauxi	generalist	21	32.5	no	11	0
Quiroga, M. F., M. I., Bor	2011	Population diet variati	Amphibia-Reptilia	32	2	26-265	Argentina	-22.26666	-64.683333	no	Hyloidea	Bufonidae	Melanophryniscus	rubiventris	generalist	41	38	no	12	0
Quiroga, M. F., M. I., Bor	2011	Population diet variati	Amphibia-Reptilia	32	2	26-266	Argentina	-22.4833333	-64.75	no	Hyloidea	Bufonidae	Melanophryniscus	rubiventris	generalist	37	38	no	14	0
Quiroga, M. F., M. I., Bor	2011	Population diet variati	Amphibia-Reptilia	32	2	26-267	Argentina	-24.0166666	-65.383333	no	Hyloidea	Bufonidae	Melanophryniscus	rubiventris	generalist	60	38	no	3	0
Norval, G., S. C. Huang, J	2014	Notes on the diets of five amphibian species fr		30		69-78	Taiwan	23.43283333	120.485	no	Ranoidea	Microhylidae	Microhyla	fissipes	generalist	39	20.4	no	15	0
Norval, G., S. C. Huang, J	2014	Notes on the diets of five amphibian species fr		30		69-79	Taiwan	23.43283333	120.485	no	Ranoidea	Microhylidae	Microhyla	heymonsi	generalist	20	21.2	no	15	0
Dan Coga' Iniceanu1, Mi	2000	Feeding in anuran con	Amphibia-Reptilia	22			Romania	45.166667	27.933333	no	Pelobatoidea	Pelobatidae	Pelobates	fuscus	generalist	170	65	no	9	0
Covaciu-Marcov, S. D., D	2010	Human influence or na	Acta Zoologica Bulgarica	62	3	307-313	Romania	44.775114	28.890118	no	Pelobatoidea	Pelopatidae	Pelobates	syriacus	generalist	32	80	no	9	0
Ghiurc?, D., & Zaharia, L.	2006	Data regarding the tro	Analele Universit??ii din	13		29-31	Romania	46.626684	26.891992	no	Ranoidea	Ranidae	Pelophylax	esculentus	generalist	104	88.8	no	9	0.003425
Guidali, F., S. Scali and A	2000	Diet and trophic niche overlap of two ranid spe		67	1	67-72	Italy	45.599756	9.532601	no	Ranoidea	Ranidae	Pelophylax	esculentus	generalist	126	88.88	no	9	0
FEREN?I, S., COVACIU-M	2009	The comparative analy	Herpetologica	3		31-39	Romania	46.403294	22.170181	no	Ranoidea	Ranidae	Pelophylax	esculentus	generalist	147	88.8	no	7	0
Paunovic, A., O. Bjelic-Ca	2010	The diet of water frog	Archives of Biological Sc	62	3	799-808	Serbia	45.134164	20.096886	no	Ranoidea	Ranidae	Pelophylax	lessonae	generalist	90	91	no	9	0
Wu, Z., Y. Li and Y. Wang	2007	A comparison of stom	Acta Zoologica Sinica	53	2	364-372	China	29.843852	122.032164	no	Ranoidea	Ranidae	Pelophylax	nigromaculatus	generalist	36	70.89	no	13	0
Burghelea, C. I., D. G.ÿZa	2010	Dietary overview of Pe	The Herpetological Jour	20	4	219-224	Spain	41.767778	0.202222	no	Ranoidea	Ranidae	Pelophylax	perezi	generalist	151	77.17	no	6	0
Balint, N., C. Indrei, R. Ia	2010	Onÿtheÿdietÿofÿthe Pe	South Western Journal c	1	1	57 - 66	Romania	44.886874	23.397387	no	Ranoidea	Ranidae	Pelophylax	ridibundus	generalist	31	145	no	10	0
Balint, N., L. Citrea, A. M	2008	Feeding ecology of the	Biharean Biologist	2		27-37	Romania	44.015991	27.776626	no	Ranoidea	Ranidae	Pelophylax	ridibundus	generalist	100	49.2	no	7	0
David N., N. Dimancea, F	2008	The analysis of the tro	Herpetologica	2		21-26	Romania	45.205613	29.325471	no	Ranoidea	Ranidae	Pelophylax	ridibundus	generalist	43	80.1	no	10	0
PeltzerÿP. M., R. C. Lajm	2000	Diet of Phyllomedusa	Bulletin de la Soci,t, her	94		11-May	Argentina	-27.45	-58.992222	no	Hyloidea	Hylidae	Phyllomedusa	hypochondrialis	generalist	25	32.31	no	42	0
de Paula Lima, J. E., D. R	2010	Diet of two sympatric	North-Western Journal c	6	1	13-24	Brazil	-14.796	-39.172	no	Hyloidea	Hylidae	Phyllomedusa	rohdei	generalist	76	45.5	no	65	0
Araujo, M. S., D. I. Bolnic	2009	Individual-level diet va	Journal of Animal Ecolog	78	4	848-856	Brazil	-18.92	-48.285	no	Hyloidea	Leptodactylidae	Physalaemus	cuvieri	generalist	60	30	no	50	0
Moore, J. E., & Stricklanc	1954	Notes on the food of t	American Midland Natu	52	1	221-224	Canada	51.378093	-109.996743	no	Hyloidea	Hylidae	Pseudacris	nigrita	generalist	37	29	no	4	0
Downie, J. R., E. G. Hanc	2010	The diet of the parado	Herpetological Journal	20	2	111-114	Trinidad	10.394414	-61.258466	no	Hyloidea	Hylidae	Pseudis	paradoxa	generalist	36	55	no	18	0
Dure´ MI, Kehr AI	2001	Differential exploitatic	Argent J Herpetol	35		340-343	Argentina	-27.499995	-58.749989	no	Hyloidea	Hylidae	Pseudis	paradoxa	generalist	50	39.6	no	42	0.031746
Downie, J. R., Hancock, E	2010	The diet of the parado	The Herpetological Jour	20	2	111-114	West Indie	10.422677	-61.056107	no	Hyloidea	Hylidae	Pseudis	paradoxa	generalist	36	57	no	21	0
Dur,, M. I., E. F. Schaefer	2004	Ecological aspects of d	Phyllomedusa: Journal c	3	2	121-131	Argentina	-27.5	-58.75	no	Hyloidea	Leptodactylidae	Pseudopaludicola	boliviana	generalist	36	15	no	44	0
Pengilley R. K.	1971	The food of some Aust	Journal of Zoology				Australia	-35.289	145.503	no	Hyloidea	Myobatrachidae	Pseudophryne	bibroni	generalist	29	23	no	11	0
Stojanova, A. M. and I. A	2008	Diet and trophic niche	ÿProcedings of the Anniversary Sc			181-190	Poland	53.835393	18.291413	no	Ranoidea	Ranidae	Rana	arvalis	generalist	46	60	no	11	0
Aszalós, L., Bogdan, H., K	2005	Food composition of t	North-Western Journal c	1		25-30	Romania	47.785174	23.089936	no	Ranoidea	Ranidae	Rana	arvalis	generalist	93	148	no	8	0
Hayes, M. P. and M. R. T	1985	DIET AND FEEDING-BE	Southwestern Naturalist	30	4	601-605	USA	34.481607	-120.230236	no	Ranoidea	Ranidae	Rana	aurora draytonii	generalist	35	69	no	5	0.010526
Cicort-Lucaciu, A. S., I. Sa	2011	Theÿfeeding studyÿof	South Western Journal c	2	1	35-46	Romania	47.735293	22.333277	no	Ranoidea	Ranidae	Rana	dalmatina	generalist	46	62	no	10	0
Aszalós, L., Bogdan, H., K	2005	Food composition of t	North-Western Journal c	1		25-30	Romania	47.785174	23.089936	no	Ranoidea	Ranidae	Rana	dalmatina	generalist	221	65	no	8	0
Hodisan, O. I., A. Benchi	2010	The trophic spectrum	Herpetologica Romanica	4		16-Jul	Romania	45.191451	23.778452	no	Ranoidea	Ranidae	Rana	dalmatina	generalist	27	57	no	5	0
Guidali, F., S. Scali and A	2000	Diet and trophic niche overlap of two ranid spe		67	1	67-73	Italy	45.599756	9.532601	no	Ranoidea	Ranidae	Rana	dalmatina	generalist	35	57	no	9	0
Bisa, R., S., Sfenthouraki	2007	Population density and	Journal of Biological Res	8		129-137	Greece	39.919505	21.260086	no	Ranoidea	Ranidae	Rana	graeca	generalist	37	37.8	no	8	0
Bisa, R., S., Sfenthouraki	2007	Population density and	Journal of Biological Res	8		129-137	Greece	39.941188	21.042795	no	Ranoidea	Ranidae	Rana	graeca	generalist	37	37.3	no	8	0
Stojanova, A. M. and I. A	2008	Diet and trophic niche	Procedings of the Anniversary Sci			181-190	Poland	53.835393	18.291413	no	Ranoidea	Ranidae	Rana	temporaria	generalist	54	75	no	11	0
Hodisan, O. I., A. Benchi	2010	The trophic spectrum	Herpetologica Romanica	4		16-Aug	Romania	45.191451	23.778452	no	Ranoidea	Ranidae	Rana	temporaria	generalist	34	75	no	5	0
Rodríguez V. , S. Nieto R	1997	Alimentación de las ra	Pirineos	149		91-104	Spain	41.642664	-0.848676	no	Ranoidea	Ranidae	Rana	temporaria	generalist	169	75	no	7	0
Attademo, A. M., W. Cej	2007	Phenology in the diet	Revista espa¤ola de herpeto		21	41-48	Argentina	-31.2461	-63.552	no	Hyloidea	Bufonidae	Rhinella	arenarum	generalist	62	135	no	8	0
Quiroga, L. B., E. A. Sana	2009	Size- and Sex-Depende	Journal of Herpetology	43	2	311-317	Argentina	-31.250747	-68.323977	no	Hyloidea	Bufonidae	Rhinella	arenarum	generalist	149	62.63	no	3	0
Ingaramo, M. D. R., J. L.	2012	Distribución y coment	Cuadernos de herpetolo	26	1	55-58	Argentina	-27.52222	-56.8014	no	Hyloidea	Bufonidae	Rhinella	azarai	generalist	22	75	no	31	0
Santana, A. S. and F. A. J	2007	Diet of Physalaemus c	Brazilian journal of biolc	67	1	125-131	Brazil	-12.563	-41.433	no	Hyloidea	Bufonidae	Rhinella	granulosa	generalist	64	31.9	no	39	0
Duré, M. I., A. I. Kehr, an	2009	Niche overlap and resc	Phyllomedusa	8	1	27-40	Argentina	-27.5	-58.75	no	Hyloidea	Bufonidae	Rhinella	granulosa	generalist	27	75	no	44	0
Sabagh, L. T., & A. M. Ca	2008	Feeding overlap in twc	Revista Brasileira de Zoc	25	2	247-253	Brazil	-22.467936	-42.992265	no	Hyloidea	Bufonidae	Rhinella	icterica	generalist	64	91.4	no	123	0
Strussmann, C., M. B. R.	1984	Dietÿandÿforaging mo	Journal of Herpetology	18	2	138-146	Brazil	-2.476180556	-54.95842222	no	Hyloidea	Bufonidae	Rhinella	marina	generalist	29	146	no	79	0
Lajmanovic R. C.	1994	Habitos alimentarios c	Revue d?Hydrobiolgie T	27	2	107-112	Argentina	-31.6667	-60.5	no	Hyloidea	Bufonidae	Rhinella	schneideri	generalist	20	149	no	30	0
Duré, M. I., A. I. Kehr, an	2009	Niche overlap and resc	Phyllomedusa	8	1	27-39	Argentina	-27.5	-58.75	no	Hyloidea	Bufonidae	Rhinella	schneideri	generalist	21	150	no	44	0
Jamieson, D. H. and S. E.	1996	Dietary diversity and c	Proceedings Arkansas Ac	50		75-78	USA	33.630215	-88.733674	no	Pelobatoidea	Scaphiopodidae	Scaphiopus	holbrookii	generalist	36	52	no	17	0
Jamieson, D. H. and S. E.	1996	Dietary diversity and c	Proceedings Arkansas Ac	50		75-79	USA	35.540975	-91.339827	no	Pelobatoidea	Scaphiopodidae	Scaphiopus	hurterii	generalist	48	60	no	13	0
Dure´ MI	1999	Interrelaciones en los	Cuad Herpetol	13		11/18/2015	Argentina	-27.499995	-58.749989	no	Hyloidea	Hylidae	Scinax	nasicus	generalist	56	22.9	no	42	0

Men,ndez-Guerrero, P	2001	Ecolog¡a tr¢fica de la c	Mongraphie, Pontificia Universidad Catolica del I				Ecuador	-1.099699	-75.80815	no	Hyloidea	Hylidae	Scinax	ruber	generalist	29	31.12	no	104	0
Bwong, B. A. and G. J. M	2010	Diet composition of Xe	African Journal of Ecolog	48	2	299-303	Kenya	-3.316972	38.380417	no	Pipoidea	Pipidae	Xenopus	borealis	generalist	76	77.1	no	33	0
Leavitt, D. J. and L. A. Fit	2009	Diet of Nonnative Hyla	Journal of Herpetology	43	3	541-545	Texas, USA	29.25	103.25	yes	Hyloidea	Hylidae	Hyla	cinerea	generalist	105	60	no	25	0
Silva, E. T. D., Filho, O. P.	2011	Predation of Native Ar	South American Journal	6	1	10/01/2014	Brazil	-20.948333	-42.2925	yes	Ranoidea	Ranidae	Lithobates	catesbeianus	generalist	135	135	no	63	0.07533
Hirai, T.	2004	Diet composition of in	Ecological Research	19	4	375-380	Japan	35.116667	135.866667	yes	Ranoidea	Ranidae	Lithobates	catesbeianus	generalist	82	120	no	13	0
Hirai, T.	2002	Ontogenetic change in	Ecological Research	17	6	639-644	Japan	35.116667	135.866667	yes	Ranoidea	Ranidae	Pelophylax	nigromaculata	generalist	478	50	no	13	0
Hinckley, A. D.	1963	Diet of the Giant Toad	Herpetelogica	18	4	253-259	Fiji	-17.868428	177.946567	yes	Hyloidea	Bufonidae	Rhinella	marina	generalist	100	146	no	1	0
Noriko Kidera, Nontivich	2008	Dietary Habits of the I	Pacific Science	62	3	423-430	Japan	24.403727	124.199843	yes	Hyloidea	Bufonidae	Rhinella	marina	generalist	60	117.1	no	10	0
Amaral, P. and R. Rebelo	2012	Diet of invasive clawe	Herpetological Journal	22	3	187-190	Portugal	38.77219	-9.421811	yes	Pipoidea	Pipidae	Xenopus	laevis	generalist	70	100	no	8	0.010574
Lobos, G. & Measey, G. J	2002	Invasive populations o	Herpetological Journal,	12	4	163-168.	Chile	-33.56897	-70.633083	yes	Pipoidea	Pipidae	Xenopus	laevis	generalist	24	88	no	2	0
Lobos, G. & Measey, G. J	2002	Invasive populations o	Herpetological Journal,	12	4	163-168.	Chile	-33.495099	-70.831154	yes	Pipoidea	Pipidae	Xenopus	laevis	generalist	21	88	no	2	0
Arau´jo MS, Dos Reis SF,	2007	Intrapopulation diet va	Copeia	###		855-865	Brazil	-18.916763	-48.283816	no	Hyloidea	Brachycephalidae	Ischnocnema	juipoca	generalist	107	14	no	50	0
Arau´jo MS, Dos Reis SF,	2007	Intrapopulation diet va	Copeia	###		855-865	Brazil	-18.916763	-48.283816	no	Hyloidea	Odontophrynidae	Proceratophrys	moratoi	generalist	50	23	no	50	0
Y?Y?T, S., TOSUNO?LU, I	1999	Feeding Biology in Buf	Turkish Journal of Zoolo	23	EK1	279-287	Turkey	38.347006	27.347307	no	Hyloidea	Bufonidae	Bufotes	viridis	generalist	80	62.8	possible	4	0.001066
Hassine, J. B.	2009	Dietÿof Discoglossusÿp	Bull. Soc. zool. Fr	134	###	321-332	Tunisia	33.751436	10.185236	no	Discoglossoidea	Alytidae	Discoglossus	pictus	generalist	142	70	possible	3	0.001135
Hirschfeld, M. and M.-O.	2011	The diet of the African	Salamandra	47	3	125-132	Benin	12.068389	3.168556	no	Ranoidea	Dicroglossidae	Hoplobatrachus	occipitalis	generalist	291	110	possible	17	0.008384
Dietl, J., W. Engels and N	2009	Diet and feeding beha	Journal of Natural Histor	43	23-2	1473-1483	Brazil	-19.816667	-43.95	no	Hyloidea	Leptodactylidae	Leptodactylus	fuscus	generalist	86	52.5	possible	61	0.002252
Sol,, M., I. R. Dias,ÿE. A.ÿ	2009	Diet of Leptodactylus (	ÿHerpetology Notes	2		15-Sep	Brazil	-14.79583	-39.172222	no	Hyloidea	Leptodactylidae	Leptodactylus	latrans	generalist	117	91.33	possible	65	0
Fran‡a, L., K.ÿFacure,ÿan	2004	Trophic and spatial nic	Studies on Neotropical F	39	3	243-248	Brazil	-18.916666	-48.2833333	no	Hyloidea	Leptodactylidae	Leptodactylus	latrans	generalist	82	100	possible	50	0.015957
Maneyro, R., D. E. Naya,	2004	Diet of the South Ame	Iheringia. S,rie Zoologia	94	1	57-61	Uruguay	-34.788184	-55.376458	no	Hyloidea	Leptodactylidae	Leptodactylus	latrans	generalist	143	70	possible	43	0.002588
Luría Manzano, R.	2012	Ecología trófica del en	Tesis - Universidad Autonoma Del Estado De Hid				Mexico	18.4	-96.845	no	Ranoidea	Ranidae	Lithobates	vaillanti	generalist	64	80.1	possible	10	0.008746
Paunovic, A., O. Bjelic-Ca	2010	The diet of water frogs	Archives of Biological Sc	62	3	799-808	Serbia	45.134164	20.096886	no	Ranoidea	Ranidae	Pelophylax	esculentus	generalist	90	77.5	possible	9	0.020161
Hirai, T. and M.ÿMatsui	1999	ÿFeeding habits of the	Copeia	4		940-947	Japan	35.1166667	135.86666	no	Ranoidea	Ranidae	Pelophylax	nigromaculatus	generalist	500	50	possible	13	0.000812
Paunovic, A., O. Bjelic-Ca	2010	Theÿdietÿofÿwaterÿfro	Archives of Biological Sc	62	3	799-808	Serbia	45.134164	20.096886	no	Ranoidea	Ranidae	Pelophylax	ridibundus	generalist	90	120	possible	9	0.020964
Cicort-Lucaciu, A. S., D. C	2011	Feeding of two amphil	North-Western Journal (	7	2	297-304	Romania	46.894425	22.480731	no	Ranoidea	Ranidae	Pelophylax	ridibundus	generalist	127	80.1	possible	8	0
FEREN?I, S., COVACIU-M	2009	The comparative analy	Herpetologica	3		31-40	Romania	46.403294	22.170181	no	Ranoidea	Ranidae	Pelophylax	ridibundus	generalist	45	80.1	possible	7	0
Hardy, L. M. and A. C. Cr	2006	Diet of amphibians an	African Journal of Herpe	55	2	143-159	Kenya	0.609844444	36.24195833	no	Ranoidea	Ptychadenidae	Ptychadena	mascareniensis	generalist	101	55	possible	19	0.00016
Tolojanahary, N. L., Fatroandriar		Diet of the Mascarene	Malagasy Nature,	5		68-74	Madagasca	-21.261667	47.459444	no	Ranoidea	Ptychadenidae	Ptychadena	mascareniensis	generalist	190	43	possible	69	0.002475
Hirai, T.	2004	Diet composition of th	Herpetological Journal	14	3	149-152	Japan	34.7	135.85	yes	Ranoidea	Dicroglossidae	Fejervarya	limnocharis	generalist	40	55	possible	12	0.004717
Silva, E. T., E. Pinheiro do	2009	Diet of the invasive fro	South American Journal	4	3	286 - 294	Brazil	-20.75	-42.51	yes	Ranoidea	Ranidae	Lithobates	catesbeianus	generalist	129	115.62	possible	66	0.12945
Bouska C. K.	2004	Temporal changes in t	MSc Thesis. Univ. Oregon				USA	44.413394	-123.326927	yes	Ranoidea	Ranidae	Lithobates	catesbeianus	generalist	395	125	possible	5	0.020636
Glorioso, B. M., J. H.ÿWa	2012	Diet of the invasive Cu	Caribbean Journal of Sci	46	###	346-355	USA	25.157851	-80.917375	yes	Hyloidea	Hylidae	Osteopilus	septentrionalis	generalist	767	50	possible	15	0.03607
Owen, J. L.ÿTexas Tech U	2005	The Cuban tree frog (Osteopilus septentrionali:	PhD dissertation,Texas T				Virgin Islan	18.437729	-64.611285	yes	Hyloidea	Hylidae	Osteopilus	septentrionalis	generalist	428	66.75	possible	5	0.002747
Bailey P	1976	Food of the marine toa	Wildlife Research	3		185?188	Papua New	-4.35	152.0333	yes	Hyloidea	Bufonidae	Rhinella	marina	generalist	162	146	possible	12	0.000825
Teixeira, R. L. and R. B. F	2010	Diet and fecundity of S	Revista espa¤ola de herpeto		24	19-25	Brazil	-20.766667	-40.583333	no	Hyloidea	Leptodactylidae	Leptodactylus	latrans	generalist	57	70	yes	74	0.027778
Ugarte, C. A., K. G. Rice a	2007	Comparison of diet, re	Copeia		2	436-448	USA	26.083333	-80.673889	no	Ranoidea	Ranidea	Lithobates	grylio	generalist	667	108.94	yes	16	0.061469
Dan Coga' lniceanu1, Mi	2000	Feeding in anuran com	Amphibia-Reptilia	22			Romania	45.166667	27.933333	no	Ranoidea	Ranidae	Pelophylax	esculentus	generalist	242	84	yes	9	0.014835
Hodar, J. A., I. Ruiz, I. & I	1990	La alimentación de la r	Miscellània Zoològica	14		145-153	Spain	36.72948	-4.346251	no	Ranoidea	Ranidae	Pelophylax	perezi	generalist	150	56	yes	7	0.005386
Mollov I. A.	2008	Sex based differences	Acta Zoologica Bulgarica	60	3	277-284	Bulgaria	42.330413	24.54054	no	Ranoidea	Ranidae	Pelophylax	ridibundus	generalist	375	58	yes	7	0.027473
€icek, K. and A.ÿMermer	2007	Food Composition of t	Turkish Journal of Zoolo	31	1	83-90	Turkey	41.780294	26.637361	no	Ranoidea	Ranidae	Pelophylax	ridibundus	generalist	53	58	yes	8	0.001161
Ruchin, A. B. and M. K. R	2002	On the diet of the mar	Advances in Amphibian	7		197-205	Russia	54.859449	46.407431	no	Ranoidea	Ranidae	Pelophylax	ridibundus	generalist	101	120	yes	9	0.002191
Mollov, I., Boyadzhiev P.	2010	Trophicÿtoleÿof the M	Bulgarian Journal of Agr	16	3	298-306	Bulgaria	42.181386	24.840486	no	Ranoidea	Ranidae	Pelophylax	ridibundus	generalist	118	120	yes	7	0.003687
Cicort-Lucaciu, A. S., C., I	2013	Note on the food com	Biharean Biologist	7	1	33-36	Romania	44.617217	22.252475	no	Ranoidea	Ranidae	Pelophylax	ridibundus	generalist	56	80.1	yes	11	0.0033
Bogdan, H. V., S. D. Cova	2012	Food composition of a	Acta Zoologica Bulgarica	64	3	253-262	Romania	45.762511	20.892783	no	Ranoidea	Ranidae	Pelophylax	ridibundus	generalist	364	80.1	yes	9	0.000367
Bull, E. L.	2003	Diet and prey availabil	Northwest Science	77	4	349-356	USA	45.093187	-118.398839	no	Ranoidea	Ranidae	Rana	luteiventris	generalist	296	87.5	yes	5	0.002309
Peterson T. Leivas1, Ferr	2012	Diet and trophic niche	Zoologia	29	5	405-412	Brazil	-25.27249722	-49.01048333	yes	Ranoidea	Ranidae	Lithobates	catesbeianus	generalist	181	125	yes	69	0.041298
Leivas, P. T., F. W. T. Leiv	2012	Diet and trophic niche	Zoologia	29	5	405-412	Brazil	-25.313056	-48.968611	yes	Ranoidea	Ranidae	Lithobates	catesbeianus	generalist	158	150	yes	70	0.078652
Krupa, J. J.	2002	Temporal shift in diet i	Southwestern Naturalist	47	3	461-467	USA	32.109573	-104.471233	yes	Ranoidea	Ranidae	Lithobates	catesbeianus	generalist	49	125	yes	13	0.001408
Boelter, R. A. and S. Z. Ce	2012	Impact of the bullfrog	Natureza & Conservacao	5	2	115-123	Brazil	-29.44722222	-53.280555	yes	Ranoidea	Ranidae	Lithobates	catesbeianus	generalist	291	125	yes	33	0.109319
Jancowski, K. and S.ÿOrc	2013	Stomach contents fror	NeoBiota	16		17-37	Canada	48.514839	-123.464096	yes	Ranoidea	Ranidae	Lithobates	catesbeianus	generalist	5075	125	yes	4	0.007176
Quiroga, L. B., Moreno, I	2015	Diet composition of ar	Journal of Natural History, (ahead-of-print), 1-14				Argentina	-31.2515	-69.4417	yes	Ranoidea	Ranidea	Lithobates	catesbeianus	generalist	169	93	yes	1	0.005405
Reed, R. N., K. A. Bakkeg	2007	Diet composition of th	Pacific Conservation Bio	13	3	219-222	Mariana Isl	14.181711	145.219873	yes	Hyloidea	Bufonidae	Rhinella	marina	generalist	336	64.2	yes	1	0.004376
Measey, G. J.	1998	Diet of feral Xenopus l	Journal of Zoology	246		287-298	Wales	51.475689	-3.17175	yes	Pipoidea	Pipidae	Xenopus	laevis	generalist	375	86.78	yes	2	0
Faraone, F. P., F.ÿLillo,ÿG	2008	The large invasive pop	Amphibia-Reptilia	29	3	405-412	Italy	37.981944	13.1225	yes	Pipoidea	Pipidae	Xenopus	laevis	generalist	59	88	yes	6	0
McCoid, M. J. & Fritts, T.	1980	Notes on the Diet of a	The Southwestern Natu	25	2	272-275	USA	33.490475	-116.969072	yes	Pipoidea	Pipidae	Xenopus	laevis	generalist	81	88	yes	8	0
Moore, J. E., & Stricklanc	1954	Notes on the food of t	American Midland Natu	52	1	221-224	Canada	51.378093	-109.996743	no	Hyloidea	Bufonidae	Anaxyrus	hemiophrys	grassland	35	67	no	4	0
Cuello, M. E., M. T. Bello	2006	Feeding habits and the	Phyllomedusa: Journal c	5	1	67-76	Argentina	-39.25	-70.283333	no	Hyloidea	Batrachylidae	Atelognathus	patagonicus	grassland	20	37.4	no	2	0
Cuello, M. E., C. A. Úbed	2009	Seasonal trophic activi	Phyllomedusa: Journal c	8	2	135-146	Argentina	-39.016666	-70.383333	no	Hyloidea	Batrachylidae	Atelognathus	patagonicus	grassland	40	50	no	2	0
Berazategui, M., A. Cama	2007	Environmental and sea	Zoological Science	24	3	225-231	Uruguay	-31.384986	-55.458582	no	Ranoidea	Microhylidae	Elachistocleis	bicolor	grassland	159	23	no	25	0
Araujo, M. S., D. I. Bolnic	2009	Individual-level diet va	Journal of Animal Ecolog	78	4	848-856	Brazil	-18.92	-48.285	no	Ranoidea	Microhylidae	Elachistocleis	bicolor	grassland	54	27.5	no	50	0
Hirai T and M. Matsui	2000	Mirmecophagy in a Ra	Zoological Science	17		459-466	Japan	35.1	135.8666667	no	Ranoidea	Ranidae	Glandirana	rugosa	grassland	160	52	no	12	0
Savini, C.-o. K., M.-F. Chu	2004	Diet Selection in the G	Internationalÿfield biology courseÿ2004				Malaysia	4.197149	114.04445	no	Ranoidea	Ranidae	Hylarana	erythraea	grassland	25	41.6	no	19	0

Amanda Saldanha Barbo	2014	Diet of Hypsiboas lept	Herpetology Notes	7		505-508	Brazil	-29.1612	-50.09	no	Hyloidea	Hylidae	Hypsiboas	leptolineatus	grassland	41	33	no	55	0
Dure´ MI, Kehr AI	2004	Influence of microhabi	Herpetologica	60		295-303	Argentina	-27.499995	-58.749989	no	Hyloidea	Leptodactylidae	Leptodactylus	bufonius	grassland	20	44	no	42	0
Piatti, L., & F. L. Souza	2011	. Diet and resource pa	Brazilian Journal of Biolo	71	3	653-662	Brazil	-20.0861111	-56.6158333	no	Hyloidea	Leptodactylidae	Leptodactylus	elenae	grassland	27	43	no	43	0
Dure´ MI, Kehr AI	2004	Influence of microhabi	Herpetologica	60		295-303	Argentina	-27.499995	-58.749989	no	Hyloidea	Leptodactylidae	Leptodactylus	latinasus	grassland	70	28	no	42	0
Piatti, L., & F. L. Souza	2011	. Diet and resource pa	Brazilian Journal of Biolo	71	3	653-663	Brazil	-20.0861111	-56.6158333	no	Hyloidea	Leptodactylidae	Leptodactylus	podicipinus	grassland	40	48	no	43	0
Crawford, J. A., D. B. She	2009	Diet Composition and	Copeia		4	642-646	USA	35.75	-95.15	no	Ranoidea	Ranidae	Lithobates	areolatus	grassland	53	31.01	no	17	0
Duré, M. I., A. I. Kehr, an	2009	Niche overlap and reso	Phyllomedusa	8	1	27-42	Argentina	-27.5	-58.75	no	Hyloidea	Bufonidae	Melanophryniscus	cupreuscapularis	grassland	22	25	no	44	0
Isacch, J. P. & M., Barg	2002	Are bufonid toads spe	Journal of Natural Histor	36	16	2005-2013	Argentina	-37.45	-57.183333	no	Hyloidea	Odontophrynidae	Odontophrynus	americanus	grassland	67	22.5	no	8	0
Wu, Z., Y. Li and Y. Wang	2007	A comparison of stoma	Acta Zoologica Sinica	53	2	364-372	China	29.843852	122.032164	no	Ranoidea	Ranidae	Pelophylax	plancyi	grassland	35	46.97	no	13	0
Alejandro Falico, D., J. Al	2012	Interpopulation and o	Revista Mexicana De Bic	83	4	1187-1193	Argentina	-30.36323889	-60.67286667	no	Hyloidea	Leptodactylidae	Physalaemus	albonotatus	grassland	128	30.2	no	42	0
Attademo, A. M., W. Cej	2007	Feeding habits of Phys	Russian Journal of Herpe	14	1	1ÿ- 6	Argentina	-31.2461	-63.552	no	Hyloidea	Leptodactylidae	Physalaemus	biligonigerus	grassland	27	35.33	no	8	0
R”dder, D.	2011	Diet of Physalaemus b	Russian Journal of Herpe	15	2	117-121	Paraguay	-21.673963	-60.814138	no	Hyloidea	Leptodactylidae	Physalaemus	biligonigerus	grassland	57	35.6	no	30	0
Pinto, P. M.	2011	Ecologia alimentar de	Thesis - Universidade de Brasilia				Brazil	-15.571951	-47.52666	no	Hyloidea	Leptodactylidae	Physalaemus	centralis	grassland	298	31	no	54	0
Santana, A. S. and F. A. J	2007	Diet of Physalaemus ci	Brazilian journal of biolo	67	1	125-131	Brazil	-12.563	-41.433	no	Hyloidea	Leptodactylidae	Physalaemus	cicada	grassland	100	24.75	no	39	0
Pinto, P. M.	2011	Ecologia alimentar de	Thesis - Universidade de Brasilia				Brazil	-15.571951	-47.52666	no	Hyloidea	Leptodactylidae	Physalaemus	cuvieri	grassland	112	30.6	no	54	0
Araujo, M. S., D. I. Bolnic	2009	Individual-level diet va	Journal of Animal Ecolog	78	4	848-856	Brazil	-18.92	-48.285	no	Hyloidea	Leptodactylidae	Physalaemus	nattereri	grassland	65	47.5	no	50	0
Canedo, C. L. A. R. I. S. S.	2006	Diet of Pipa carvalhoi (	Herpetological Review	37	1	44-45	Brazil	-14.791923	-39.032869	no	Pipoidea	Pipidae	Pipa	carvalhoi	grassland	54	60	no	57	0
Christian KA	1982	Changes in the food ni	Copeia	###		73-80	USA	40.504061	-104.968896	no	Hyloidea	Hylidae	Pseudacris	maculata	grassland	393	22	no	8	0
Miranda, T., M. Ebner, M	2006	Spatial, seasonal and i	South American Journal	1	2	121-130	Brazil	-29.583333	-50.133333	no	Hyloidea	Hylidae	Pseudis	cardosoi	grassland	130	44.5	no	38	0.001339
Dure´ MI, Kehr AI	2001	Differential exploitatic	Argent J Herpetol	35		340-343	Argentina	-27.499995	-58.749989	no	Hyloidea	Hylidae	Pseudis	laevis	grassland	75	16.9	no	42	0
Conradie, W., W. R. Brar	2010	Notes on the diet of re	Herpetology Notes	3		215-219	South Afric	-32.556944	22.572222	no	Ranoidea	Pyxicephalidae	Pyxicephalus	adspersus	grassland	89	39.3	no	9	0.087324
Piatti, L., & F. L. Souza	2011	. Diet and resource pa	Brazilian Journal of Biolo	71	3	653-664	Brazil	-20.0861111	-56.6158333	no	Hyloidea	Bufonidae	Rhinella	bergi	grassland	50	42	no	43	0
Isacch, J. P. & M., Barg	2002	Are bufonid toads spe	Journal of Natural Histor	36	16	2005-2012	Argentina	-37.45	-57.183333	no	Hyloidea	Bufonidae	Rhinella	dorbignyi	grassland	40	20.2	no	8	0
Duré, M. I., A. I. Kehr, an	2009	Niche overlap and reso	Phyllomedusa	8	1	27-41	Argentina	-27.5	-58.75	no	Hyloidea	Bufonidae	Rhinella	fernandezae	grassland	21	75	no	44	0
Measey, G. J. andÿRoyer	2005	An examination of Pip	The Herpetological Jour	15	4	291-294	Venezuela	10.1	-68.133333	yes	Pipoidea	Pipidae	Pipa	parva	grassland	42	37.34	no	18	0
Piatti, L., & F. L. Souza	2011	. Diet and resource pa	Brazilian Journal of Biolo	71	3	653-661	Brazil	-20.0861111	-56.6158333	no	Hyloidea	Leptodactylidae	Leptodactylus	chaquensis	grassland	121	65	possible	43	0.003774
Hirai, T.ÿand M. Matsui	2001	Food habits of an enda	Ecological Research	16	4	737-743	Japan	35.1333333	135.983333	no	Ranoidea	Ranidae	Pelophylax	porosus	grassland	58	52	possible	12	0.002387
Smith, C. C., & A. N. Brag	1949	Observations on the e	Ecology	30	3	333-352	USA	35.164006	-97.207129	no	Hyloidea	Bufonidae	Anaxyrus	compactilis	shrubland	94	90	no	15	0
Brito, L., F. Aguiar and P.	2012	Diet composition and	South American Journal	7	1	55-61	Brazil	-4.267553	-38.936311	no	Hyloidea	Odontophrynidae	Odontophrynus	carvalhoi	shrubland	21	54.7	no	30	0
Pengilley R. K.	1971	The food of some Aust	Journal of Zoology				Australia	-35.289	145.503	no	Hyloidea	Myobatrachidae	Pseudophryne	corroboree	shrubland	275	30	no	11	0
Dure´ MI	1999	Interrelaciones en los	Cuad Herpetol	13		11/18/2015	Argentina	-27.499995	-58.749989	no	Hyloidea	Hylidae	Scinax	acuminatus	shrubland	42	34.8	no	42	0
Sabagh, L. T., V. L. Ferrei	2010	Living together, somet	Brazilian Journal of Biolo	70	4	955-959	Brazil	-19.56666	-57	no	Hyloidea	Hylidae	Scinax	acuminatus	shrubland	31	40.7	no	43	0
COURANT, J., THIRION, J	2014	Le régime alimentaire	Bulletin de la Société he	150			France	47.036426	-0.308333	yes	Pipoidea	Pipidae	Xenopus	laevis	generalist	23	110	no	10	0
Tolojanahary, N. L., Fatro	2011	Diet of the Mascarene	Malagasy Nature,	5		68-74.	Madagasca	-21.261692	47.459434	no	Ranoidea	Ptychadenidae	Ptychadena	mascareniensis	generalist	190	43	possible	69	0.002347
de Oliveira, E. G., & Hado	2015	Diet Seasonality and F	Journal of Herpetology	49	2	252-256	Brazil	-23.35265556	-45.13283611	no	Hyloidea	Brachycephalidae	Brachycephalus	pitanga	forest	30	11.75	no	89	0
Pizzatto, L., & R. Shine	2008	The behavioral ecolog	Behavioral Ecology and S	63	1	123-133	Australia	-12.66	131.336	yes	Hyloidea	Bufonidae	Rhinella	marina	generalist	131	146	yes	23	0.155056
Allingham, S. M. & M., H	2011	Feeding Ecology of Kas	Current Herpetology	30	2	137-143	Cameroon	8.333333333	13.83333333	no	Ranoidea	Hyperolidae	Kassina	senegalensis	generalist	55	33	no	24	0
Ferreira, R. B., K. H., Bea	2015	Diet of the Nonnative	Journal of Herpetology	49	2		Hawaii	20.740838	-156.441316	yes	Hyloidea	Eleutherodactylidae	Eleutherodactylus	planirostris	forest	397	21.1	no	5	0
Neves, M. D. O., C. J. D. I	2014	Sexual Dimorphism an	South American Journal	9	3	177-182	Brazil	-9.622019	-48.27751	no	Hyloidea	Hylidae	Pseudis	tocantins	generalist	159	45	possible	43	0.004444
Teixeira, R. L., & R. B. Fer	2010	Diet and fecundity of S	Revista española de her	24		19-25	Brazil	-20.800753	-40.65657	no	Hyloidea	Hylidae	Sphaenorhynchus	planicola	generalist	88	22.5	no	73	0
de Oliveira, M., Gottscha	2015	Diet composition and	Herpetology Notes,	8		173-177	Brazil	-32.115284	-52.210201	no	Hyloidea	Leptodactylidae	Physalaemus	gracilis	generalist	44	43	no	23	0
de Oliveira, M., Gottscha	2015	Diet composition and	Herpetology Notes,	8		173-177	Brazil	-32.115284	-52.210201	no	Hyloidea	Leptodactylidae	Physalaemus	biligonigerus	generalist	49	40	no	23	0
Raoni Rebouças & Mirco	2015	Diet of Adenomera tho	Studies on Neotropical F	http://dx.doi.org/10.108			Brazil	-13.833359	-39.166867	no	Hyloidea	Leptodactylidae	Adenomera	thomei	forest	91	20.13	no	56	0
Luiz, Contrera & Neckel-	2015	Diet and tadpole trans	Herpetological Journal				Brazil	-2.161533	-56.084417	no	Hyloidea	Dendrobatidae	Ameerega	trivittata	forest	56	38	no	76	0
Agudelo, G. A., R. S. Cast	2014	Diet of the Blue-Bellie	Journal of Herpetology	49	2		Colombia	3.977107	-77.336378	no	Hyloidea	Dendrobatidae	Andinobates	minutus	forest	150	11.8	no	40	0
Isaacs, P., & J. M., Hoyos	2010	Diet of the Cane Toad	South American Journal	5	1	45-50	Colombia	4.602777778	-75.83333333	no	Hyloidea	Bufonidae	Rhinella	marina	generalist	49	146	no	5	0
Macale D, Vignoli L, Carp	2008	Food selection strateg	Herpetological Journal,	18		49-58	Argentina	-28.475558	-57.200598	no	Hyloidea	Hylidae	Dendropsophus	nanum	generalist	47	20	no	32	0
Macale D, Vignoli L, Carp	2008	Food selection strateg	Herpetological Journal,	18		49-58	Argentina	-28.475558	-57.200598	no	Hyloidea	Hylidae	Dendropsophus	sanborni	grassland	76	16.6	no	32	0
Vignoli L, Luiselli L, Bolog	2009	Dietary patterns and o	Vie Milieu Life Environ	59		47-57	Italy	42.139649	12.109967	no	Ranoidea	Ranidae	Pelophylax	esculentus	generalist	104	88.8	no	8	0.006579
Vignoli L, Luiselli L, Bolog	2009	Dietary patterns and o	Vie Milieu Life Environ	59		47-57	Italy	42.139649	12.109967	no	Hyloidea	Hylidae	Hyla	intermedia	generalist	62	45	no	8	0
